# Less Is More: A Case of Delayed Return of Normal Conduction

**DOI:** 10.7759/cureus.104443

**Published:** 2026-02-28

**Authors:** Asher Gorantla, Varshitha Panduranga, Srivane Richard, Francesca Cali, Suzette Graham-Hill

**Affiliations:** 1 Internal Medicine, State University of New York Downstate Health Sciences University, Brooklyn, USA; 2 Cardiology, State University of New York Downstate Health Sciences University, Brooklyn, USA

**Keywords:** atrioventricular block, complete heart block, conduction recovery, extended monitoring, inferior myocardial infarction, permanent pacemaker avoidance, temporary transvenous pacing

## Abstract

Conduction disturbances are a well-recognized complication of acute myocardial infarction (MI), particularly when the infarct involves the inferior wall and jeopardizes blood flow to the atrioventricular node. An 82-year-old woman admitted for symptomatic anemia developed an inferior myocardial infarction complicated by 2:1 atrioventricular block, later progressing to complete heart block. She underwent percutaneous intervention for a critical distal right coronary artery lesion and required temporary transvenous pacing. Although permanent pacemaker implantation was initially considered, the procedure was delayed due to concurrent management of hemorrhagic cystitis, allowing for extended observation. During this period, her native atrioventricular conduction fully recovered, and she remained stable after removal of the temporary pacemaker, with an implantable cardiac monitor placed for follow-up. This case demonstrates that conduction recovery after inferior MI may occur beyond the standard 72-hour window and supports prolonged monitoring to avoid unnecessary pacemaker implantation in appropriate patients.

## Introduction

Atrioventricular (AV) conduction disturbances are a frequent complication of acute myocardial infarction (MI), particularly when the infarct involves the inferior wall and compromises blood flow to the AV node [1-3]. In inferior MI, high-grade AV block is typically transient and nodal in origin, with recovery of AV conduction reported in approximately 70-90% of patients within 24-72 hours following reperfusion therapy [4]. Although many patients recover conduction within the first few days after reperfusion, the timing can be unpredictable, and premature decisions about permanent pacing may expose patients to avoidable long-term device-related risks [2]. Current guidelines recommend a 72-hour observation period before committing to pacemaker implantation; however, clinical experience suggests that recovery can occur beyond this window in selected cases [5]. This report describes an elderly patient with inferior MI and complete heart block (CHB) whose conduction unexpectedly returned after an extended period of temporary pacing, emphasizing the importance of individualized decision-making when managing post-infarction bradyarrhythmia [2,4].

## Case presentation

An 82-year-old woman was initially admitted for management of symptomatic anemia. On presentation, her hemoglobin was 6 g/dL (reference range 12.0-16.0 g/dL), which improved after receiving packed red blood cell transfusions. During her hospitalization, the patient developed acute, severe substernal chest pain radiating to the left arm, associated with diaphoresis. Her past medical history included hypertension and hyperlipidemia treated medically, and hemorrhagic cystitis managed with urologic interventions. She had no history of prior coronary artery disease, prior heart blocks or pacemaker implantation, and no history of thyroid disease. Her medication history included hydrochlorothiazide for blood pressure and atorvastatin for hyperlipidemia. She denied any use of beta blockers or other atrioventricular (AV) nodal blocking agents. 

**Figure 1:**
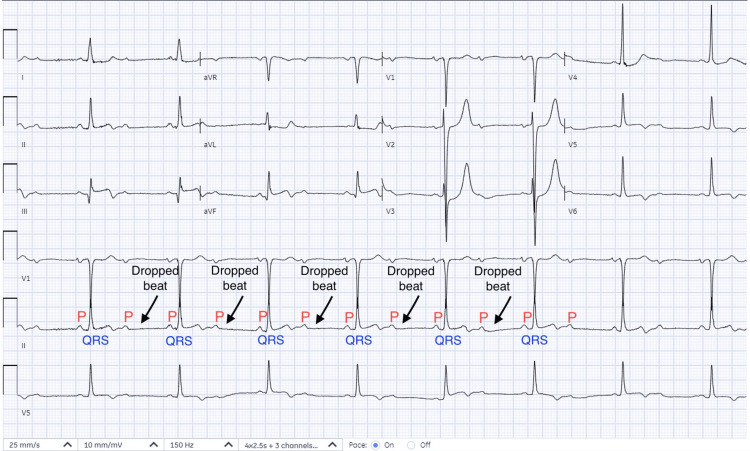
Electrocardiography (EKG) showing sinus rhythm with inferior wall MI and 2:1 AV block. MI: myocardial infarction; AV: atrioventricular.

Initial laboratory evaluation revealed a hemoglobin level of 6 g/dL (reference range 12.0-16.0 g/dL), which improved following red blood cell transfusions. Cardiac biomarkers demonstrated a markedly elevated troponin I level of 5 ng/mL (reference range 0.03-0.04 ng/mL). Serum electrolytes and renal function testing were within normal limits. Thyroid-stimulating hormone (TSH) of 1.2 mIU/L (reference range 0.4-4.0 mIU/L). Electrocardiography (Figure 1) showed ST-segment elevation in the inferior leads with a high-grade 2:1 AV block.

**Figure 2:**
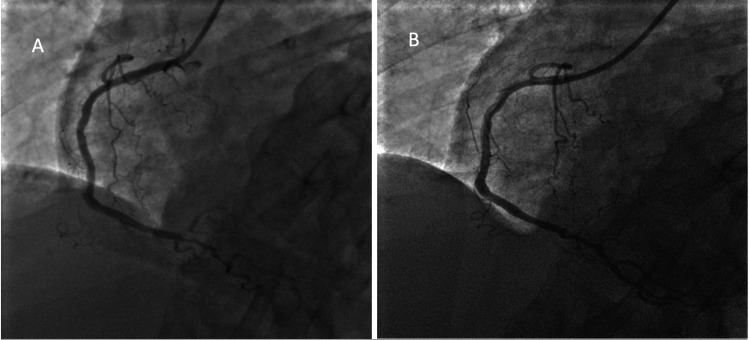
Coronary angiography showing (A) 99% stenosis of the distal RCA at the ostium of the PDA and (B) with culprit lesion successfully treated with PCI using a Resolute Onyx DES 2.25 x 12 mm. RCA: right coronary artery; PCI: percutaneous coronary intervention; PDA: posterior descending artery; DES: drug-eluting stent.

Transthoracic echocardiography demonstrated a preserved left ventricular ejection fraction of 60%. The patient underwent coronary angiography (Figure 2), which identified a 99% stenosis of the distal right coronary artery and was successfully treated with percutaneous coronary intervention (PCI) and stent placement. 

**Figure 3:**
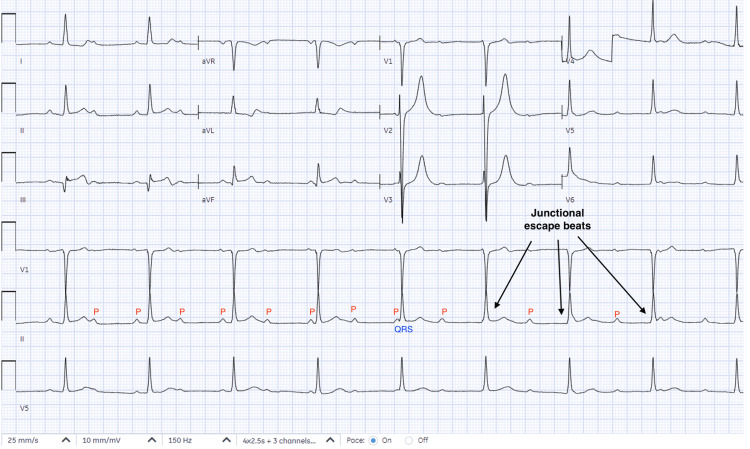
EKG showing CHB post-revascularization on day two. EKG: electrocardiography, CHB: complete heart block.

Following PCI, the patient remained in complete heart block (CHB) (Figure 3). 

**Figure 4:**
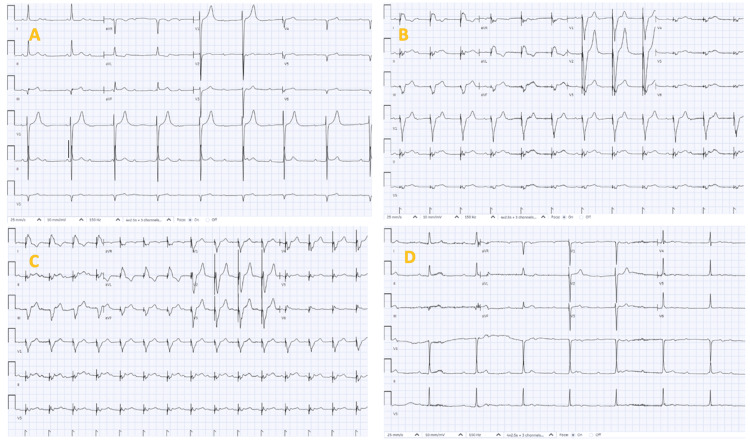
Serial electrocardiographs post-revascularization showing (A)-(D) days three to six, still in CHB and ventricularly paced, seen in (B) and (C). CHB: complete heart block.

Intravenous atropine 0.5 mg was administered without improvement in heart rate. Additional doses were deferred, given the concern for possible worsening of coronary ischemia in the setting of acute myocardial infarction. A temporary transvenous pacemaker (TVP) was placed for bradycardia management, and she was monitored in the cardiac intensive care unit. After 72 hours, she remained in CHB. Permanent pacemaker (PPM) implantation was planned but delayed due to ongoing management of hemorrhagic cystitis, which required urologic procedures including cystoscopy and continuous bladder irrigation, with continued need for blood transfusions. Given the patient's hemodynamic stability and reversible ischemic etiology, the decision was made to extend the observation period with the TVP in place. The TVP was set at a rate of 60 beats per minute with an output of 3 mA. These parameters were not changed during her hospital course. Remarkably, after seven days, native AV conduction recovered spontaneously (Figure 4). 

**Figure 5:**
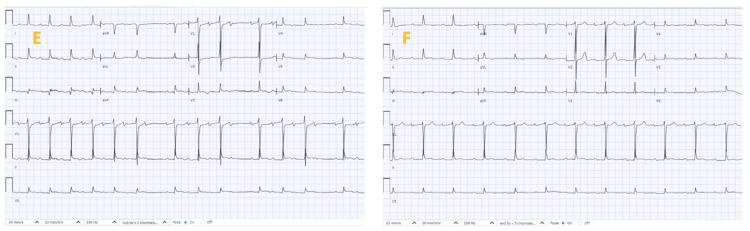
Serial electrocardiographs showing (E) day seven post-revascularization in sinus rhythm with Mobitz type 1 AV block and (F) day eight post-removal of TVP remained in normal sinus rhythm. TVP: temporary transvenous pacemaker.

Following removal of the TVP, the patient remained in normal conduction (Figure 5).

The patient remained asymptomatic with normal conduction throughout her hospital course following TVP removal. An implantable cardiac monitor was inserted prior to discharge for continuous monitoring for heart blocks. The patient was seen in the outpatient cardiology clinic six weeks after discharge and continued to remain in sinus rhythm, with no AV blocks detected upon device interrogation.

## Discussion

Conduction disturbances are a well-recognized complication of acute myocardial infarction (MI), particularly inferior wall infarction. High-grade atrioventricular (AV) block occurs in approximately 10-15% of inferior ST-elevation MIs and is usually related to reversible nodal ischemia rather than permanent conduction system necrosis [1,2]. In most individuals, the AV node is supplied by the AV nodal branch of the right coronary artery (RCA); acute RCA occlusion therefore produces transient ischemia of nodal tissue. Unlike anterior MI, where AV block often reflects extensive septal injury and carries a poor prognosis, inferior MI-associated AV block is typically nodal, vagally mediated, and reversible with reperfusion [1,3].

The pathophysiology of conduction recovery is dynamic. Restoration of blood flow via percutaneous coronary intervention (PCI) may normalize conduction immediately, but recovery can be delayed due to tissue edema, metabolic derangement, and reperfusion injury. Observational data demonstrate that AV block after inferior MI may resolve over several days despite persistent early complete heart block (CHB) [2]. Because nodal tissue may remain viable even when electrically silent, the absence of early recovery does not necessarily imply irreversible injury.

The 2018 American College of Cardiology (ACC)/American Heart Association (AHA)/Heart Rhythm Society (HRS) Guideline on Bradycardia and Cardiac Conduction Delay specifically recommends observation before permanent pacemaker (PPM) implantation in patients with AV block following acute MI when a reversible ischemic cause is suspected [4]. The guideline emphasizes that temporary pacing alone is not an indication for permanent pacing and that decisions should incorporate infarct location, likelihood of recovery, and overall clinical stability. In inferior MI, permanent pacing is reserved for persistent advanced second-degree or third-degree AV block not expected to resolve after an adequate observation period. Importantly, the document highlights that recovery may extend beyond the conventional 72-hour window in selected patients [4].

These principles are reinforced by the 2023 ACC/AHA/American College of Clinical Pharmacy (ACCP)/HRS Atrial Fibrillation Guideline, which reiterates that potentially reversible causes of bradyarrhythmia should be addressed before committing to long-term device therapy and that pacing decisions should prioritize reversibility and patient-specific risk assessment [5]. Although focused on atrial fibrillation, the 2023 guideline underscores a broader contemporary shift toward conservative, physiology-guided device implantation.

In addition to ischemia, alternative reversible contributors to AV block must be considered. Severe anemia, heightened vagal tone related to pain or bleeding, medication effects, and electrolyte abnormalities may exacerbate conduction delay. In our case, the electrolytes and thyroid-stimulating hormone levels were within normal limits. The patient was also not on any AV nodal blocking medications. However, the physiologic stress from hemorrhagic cystitis and anemia may have contributed to a transient autonomic imbalance. Hence, recognition and correction of these factors further supported an extended period of observation.

Pharmacologic therapy may be attempted in the acute setting. Atropine is recommended for symptomatic bradycardia due to AV nodal block and may transiently improve conduction when increased vagal tone contributes to the disturbance [5,6]. However, in ischemic high-grade AV block-particularly when related to structural nodal hypoperfusion-atropine is frequently ineffective, as parasympathetic blockade does not correct underlying ischemic injury [3,5]. In our patient, atropine 0.5 mg was administered after PCI without improvement in heart rate or AV conduction. Additional doses were deferred, given the absence of response and concerns that repeated atropine administration may increase myocardial oxygen demand and potentially exacerbate ischemia in the acute infarction setting [5,7]. Temporary pacing was, therefore, prioritized for reliable chronotropic support.

Temporary transvenous pacing served as a bridge to maintain hemodynamic stability while allowing time for myocardial recovery. The decision to continue temporary pacing beyond seven days was guided by several clinical considerations: the patient demonstrated intermittent intrinsic conduction, suggesting viability of the AV node; she remained hemodynamically stable without pacing dependency, and immediate PPM implantation would have carried elevated procedural risk in the setting of active urologic bleeding. While prolonged temporary pacing carries risks -- including infection, venous thrombosis, and lead displacement -- these risks were judged lower than the lifelong implications of premature permanent pacing.

Permanent pacemaker implantation, although generally safe, is associated with complication rates of approximately 3-7%, including infection, lead dislodgement, venous occlusion, and procedure-related mortality [8,9]. Long-term device therapy also entails generator replacements, lead failure risk, and psychosocial burden. Careful identification of patients likely to recover intrinsic conduction is therefore essential to minimize avoidable device exposure.

Notably, our patient demonstrated complete recovery of AV conduction after approximately one week. At three-month follow-up, implantable cardiac monitor interrogation showed a stable sinus rhythm without recurrence of high-grade block. This sustained recovery supports the decision for extended observation and illustrates the unpredictable yet reversible nature of ischemic nodal dysfunction.

Overall, this case highlights that inferior MI-related AV block is often transient and that pacing decisions should integrate guideline recommendations with individualized clinical judgment. Extended monitoring in stable patients with suspected reversible ischemia may prevent unnecessary permanent pacemaker implantation and aligns with a patient-centered approach to post-infarction bradyarrhythmia management [2,4].

## Conclusions

This case emphasizes the need for individualized pacing decisions following inferior wall MI-associated heart block. While guidelines recommend a 72-hour observation period before permanent pacing, clinicians should remain vigilant for late recovery of AV conduction beyond this window. Careful monitoring with temporary pacing and noninvasive follow-up using implantable monitors can prevent unnecessary permanent pacemaker implantation and its associated risks.
